# Upcycling of black currant pomace for the production of a fermented beverage with *Wolfiporia cocos*

**DOI:** 10.1007/s13197-023-05677-4

**Published:** 2023-02-06

**Authors:** Svenja Sommer, Janine Laura Hoffmann, Marco Alexander Fraatz, Holger Zorn

**Affiliations:** 1 Institute of Food Chemistry and Food Biotechnology, Justus Liebig University Giessen, Heinrich-Buff-Ring 17, 35392 Giessen, Germany; 2 Fraunhofer Institute for Molecular Biology and Applied Ecology, Ohlebergsweg 12, 35392 Giessen, Germany

**Keywords:** Lemonade, Side stream, Pomace, Fungus, Basidiomycota

## Abstract

Pomace as a side stream from black currant juice production is mostly discarded, even though it is rich in nutrients like protein, fiber, sugars, anthocyanins, polyphenols, and other secondary metabolites. Fungi from the division of Basidiomycota have a great enzymatic toolbox to recycle these complex mixtures of nutrients. In particular, the edible medicinal fungus *Wolfiporia cocos* has been described as a suitable biocatalyst to form pleasant aroma compounds in fermentation processes. Therefore, medium optimization, upscaling, and filtration were performed to produce a beverage based on black currant pomace fermented with *W. cocos*. A trained panel described the beverage as highly pleasant, reminiscent of honey, flowers and berries with a well-balanced sour and sweet taste. The flavor compounds linalool (citrus), geraniol (flowery), phenylacetic acid (honey), methyl phenylacetate (honey), eugenol (clove), and 2-phenylethanol (rose) were produced during fermentation and the concentrations exceeded their respective odor thresholds. The produced beverage was evaluated with 8.0 ± 1.4 from 10 for the question of whether panelists would buy the product. Fungal fermentation with the edible fungus *W. cocos* enabled the production of a highly pleasant beverage and additionally may reduce waste by using pomace and table sugar as sole ingredients.

**Supplementary Information:**

The online version contains supplementary material available at 10.1007/s13197-023-05677-4.

## Introduction

In a situation of depriving resources, great needs for sustainable solutions for feeding the growing population arise. Recycling of food side streams becomes thus more and more important. Pomace, as a pressing residue of the juice industry, represents a readily available and highly interesting side stream. Pomace mainly consists of fruit skin, kernels, pulp, and juice residues. As it is rich in fiber, protein, valuable unsaturated fatty acids, dyes, vitamins, and phytochemicals, pomace can contribute to a healthy diet (Farooque et al. 2018; Struck et al. 2016). Particularly, potentially healthy nutrients are anthocyanins, flavonoids, tannins, phenolic acids, and hydroxycinnamates, which may have a positive influence on cardiovascular-associated, ocular, and neurodegenerative neoplastic diseases (Farooque et al. 2018; Gopalan et al. 2012). Black currant pomace is typically characterized by a very low pH value of 2.6–2.8. This ensures a comparatively high microbiological stability, but complicates its recycling by traditional fermentation processes (Bagger-Jørgensen and Meyer 2004). Attempts to use pomace as a substitute for flour in baking products, as an ingredient in smoothies, as a source of antioxidants, or as a substrate for alcoholic fermentation have been published (May and Guenther 2020; Rohm et al. 2015; Struck et al. 2016). Nevertheless, it is still predominantly used for composting, biogas production, as feed or burned for power generation (May and Guenther 2020; Rohm et al. 2015).

Utilization of this side-stream has a great potential for saving resources, improving the efficiency of crop growing, and reducing costs (May and Guenther 2020). As black currant spritzer is a popular drink, its pomace may be particularly suitable for use in novel beverages. Fermented beverages have been consumed for ages as fermentation may improve taste, texture, nutrients, and shelf life (Hugenholtz 2013). Alcohol-free fermented products are traditionally associated with health benefits and are currently becoming more and more popular. Examples of alcohol-free beverages, which are fermented by bacteria and yeasts, comprise, *e.g.* Kombucha, Kvass, water kefir, and *Bionade* (Hugenholtz 2013; Marsh et al. 2014). In recent studies, microorganisms like *Lactobacillus* species act as an additive for functional beverages with proposed health benefits (Hashemi et al. 2021; Jouki et al. 2021). Furthermore, some rare examples of beverages produced with higher fungi have been published. Besides using *Trametes versicolor*, *Lentinula edodes*, and *Flammulina velutipes*, *W. cocos* was successfully used to biotransform tea or wort leading to a highly pleasant flavor (Rigling et al. 2021a, 2021b, 2022; Wu et al. 2016; Zhang et al. 2014, 2015). These fungi belong to the division of Basidiomycota, which are known for their unique properties to utilize nutrients. As they can degrade cellulose and hemicellulose, they can use substrates, which are otherwise hard to upcycle (Bouws et al. 2008). Especially *W. cocos* has been reported to be a highly suitable biocatalyst to produce pleasant aromas and fruit acids. It is an edible medicinal mushroom, which is also known in China as *Fu Ling* or Indian Bread (Wang et al. 2013). Highly fragrant compounds like linalool, methyl anthranilate, phenyl ethanol, benzaldehyde, methyl phenylacetate, and geraniol have been detected in several studies (Rigling et al. 2021a; Sommer et al. 2021; Wu et al. 2016).

In this study, *W. cocos* was used as a biocatalyst to upcycle black currant pomace for the beverage industry. Suitable culture conditions to produce a pleasant taste and odor from black currant pomace were identified, and relevant aroma compounds and fruit acids were identified and quantified.

## Materials and methods

### Chemicals

Chemicals were obtained from the following suppliers: acetone (99.8%) from Acros Organics B.V.B.A., sodium carbonate (≥ 99%, water free), sulfuric acid (98%), d-glucose, malt extract, and sodium hydroxide (≥ 98%) from Carl Roth GmbH & Co. KG (Karlsruhe, Germany), d-fructose from Hamburger Zuckergesellschaft mbH (Hamburg, Germany), sucrose from Südzucker (Mannheim, Germany), and sodium hydrogen carbonate (≥ 99.7%) from Th. Geyer GmbH & Co. KG (Renningen, Germany).

Authentic standard compounds were purchased from commercial sources: geraniol (99%), malic acid (> 99%), 2-nonanone (99%), 1-octen-3-ol (98%), 2-phenylethanol (99%), and terpinen-4-ol (97%) from Acros Organics B.V.B.A (Fair Lawn, USA), phenylacetic acid (99%) from Alfa Aesar (Haverhill, USA), acetic acid (100%), citric acid (water free), and oxalic acid-dihydrate from AppliChem GmbH (Darmstadt, Germany), l-tartaric acid (≥ 99.5%), eugenol (pure) from Carl Roth GmbH, linalool (97%), methyl phenylacetate (> 99%), *trans*-nerolidol (analytical standard), 2-nonanol (99%), and lactic acid (≥ 85%) from Sigma Aldrich (St. Louis, USA), and 3,4-dimethylbenzaldehyde (95%) from TCI Chemicals (Tokyo, Japan).

### Fungus and substrate

*W. cocos* (No. 279.55) was purchased from CBS Fungal Biodiversity Centre (Utrecht, Netherlands). Frozen black currant pomace of the cultivar *Ribes nigrum* L. cv. Tihope was provided by the Department for Pomology, Geisenheim University (Geisenheim, Germany).

### Fermentation and medium optimization

Fermentations were performed under similar conditions as described in the literature (Rigling et al. 2021b; Zhang et al. 2014, 2015) *W. cocos* was maintained on malt extract agar (20 g L^−1^ malt extract, 15 g L^−1^ agar–agar). All media were prepared with drinking water and autoclaved at 121 °C for 20 min. As pre-culture, 200 mL ME medium (20 g L^−1^ malt extract) in a 500 mL Erlenmeyer flask was inoculated with an overgrown agar plug (diameter 0.4 cm) and homogenized using an Ultra Turrax homogenizer (T25, IKA-Werke GmbH & CO. KG, Staufen, Germany) for 30 s with 10,000 rpm. After incubation for 7 days at 24 °C with shaking at 150 rpm in the dark, the pre-culture was homogenized and centrifuged for 5 min at 10 °C at 3,500 g. The supernatant was disposed and replaced with autoclaved drinking water. Main cultures were inoculated with 10% (v/v) of the washed pre-culture and cultivated with 150 rpm at 24 °C in the dark.

For medium optimization, pomace concentrations of 60 g L^−1^, 80 g L^−1^, 100 g L^−1^, and 120 g L^−1^ were evaluated. The pH-value was adjusted to 4.4 before autoclaving with NaOH. pH measurements were performed with a Five Easy™ pH bench meter FE 20 (Mettler-Toledo, Columbus, USA), which was calibrated each day using buffers of pH 2, 4, and 7 pursued from Carl Roth GmbH+ Co. KG. Sugar solutions were autoclaved separately and added to the medium before inoculation in equally sweet concentrations of 100 mL L^−1^ sucrose-syrup (580 g L^−1^ sucrose), 89 mL L^−1^ fructose-syrup (574 g L^−1^ fructose) and 167 mL L^−1^ glucose-syrup (502 g L^−1^). 75 mL L^−1^ sucrose syrup were added to test reduced sugar content. The final medium composed of 80 g L^−1^ black currant pomace and 100 mL L^−1^ sucrose-syrup (corresponding to 58 g L^−1^ sucrose) was fermented for three to four days until a pH 3.5 ± 0.1 was reached. The culture volume was scaled-up from 0.2 L in 0.5 L Erlenmeyer flasks (EF), 0.8 L in 2 L EF, 2 L in 5 L EF to 4 L in a 7.5 L fermenter (Labfors 3, Infors GmbH, Einsbach, Germany). The process parameters for the fermenter were 150 rpm (propeller stirrer), 1 L min^−1^ air vent flow during the first three hours, 0.3 L min^−1^ air vent flow afterwards, and 24 °C. The pH was continuously measured with a pH electrode (405-DPAS-SC-K8 S/120, Mettler Toledo), which was calibrated as reported above. After fermentation, mycelium and other solid matter were removed with a cheesecloth (100% cotton). The beverage was carbonated under pressure with 1.7 bar of food grade carbon dioxide (2.5, Praxair Deutschland GmbH) for three days at 4 °C.

**Fig. 1:**
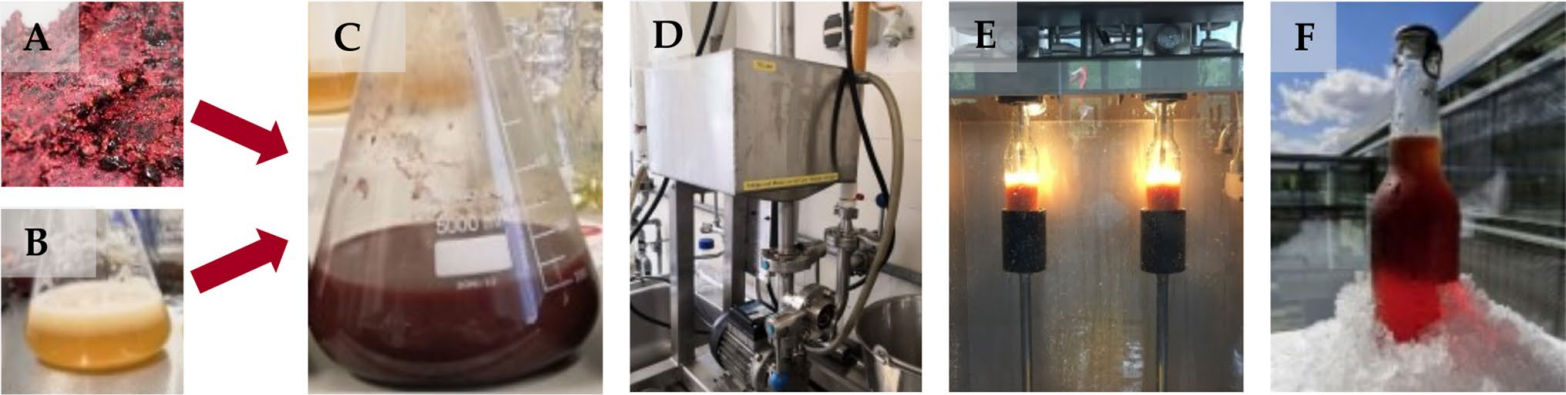
Processing of the beverage: black currant pomace (**A**), pre-culture of *W. cocos* in malt extract medium (**B**), fermentation in shaking flasks (**C**), cross-flow filtration (**D**), filling into bottles (**E**), and produced beverage (**F**)

To investigate the influence of microfiltration and pasteurization, 15 L of the fermented beverage was produced in 5 L EF containing 2 L medium. Prior to filtration, the beverage was treated with 0.67 mL L^−1^ of Fructozym Flow UF (Erbslöh Geisenheim GmbH; Geisenheim, Germany) for 3 h at 4 °C. For cross-flow filtration, the beverage was cooled and treated with a Romicon Hollow Fiber Cartridge (100 µm, Koch Separation Solutions, Massachusetts, USA) at 2 bar. The permeate was short time-pasteurized for a few seconds at 78 °C, cooled down to 4 °C, and carbonated for three days (5 g L^−1^ CO_2_). Afterwards, the sparkling beverage was filled into 0.33 L bottles with the Juice dispenser PAS1-PAS2-81-V2 (mabo Steuerungselemente GmbH, Epplingen, Germany) and pasteurized (45 min, 78 °C) (Fig. 1).

### Analysis of fruit acids and sugars

Sugars were quantified with the sucrose/d-fructose/d-glucose assay kit from Megazyme Ltd. (Wicklow, Ireland). Fruit acids were analyzed using an ion chromatograph with a conductivity detector (883 Basic IC Plus, Deutsche METROHM GmbH & Co. KG, Filderstadt, Germany). For analysis of acetic acid, citric acid, lactic acid, malic acid, and tartaric acid, a Metrosep organic acids column (250 · 7.8 mm, Metrohm) and a pre-column (Metrosep Organic Acids Guard, 50 · 4.6 mm, Metrohm) were used. The eluent consisted of 0.5 mM H_2_SO_4_ in deionized water/acetone (85/15, v/v) with a constant flow of 0.5 mL min^−1^. The injection volume was 20 µL. Samples were diluted with deionized water (1:8), filtered (PET, 0.45 µm, MACHEREY–NAGEL, Düren, Germany), quantified with external calibration, and identified by addition of the respective standards. Oxalic acid was quantified with a Metrosep A sup 4-350 column (250 · 4.0 mm, Metrohm) using an eluent of 1.8 mM Na_2_CO_3_ and 1.7 mM NaHCO_3_ in deionized water/acetone (98/2, v/v) and a flow of 1 mL min^−1^. The suppressor solution consisted of 50 mM H_2_SO_4_ in deionized water. Samples were diluted with pure water (1:40), filtered (0.45 µm), identified by addition of the standard, and quantified by external calibration.

### Sensory analysis

For sensory analysis by a trained panel (*n* = 20, 10 male, 10 female, non-smokers, 17–44 years old), beverages were served at 4 °C in 40 mL glasses. Experiments were performed at white light. Each participant received 20 mL of the beverages and was asked to rate odor, smell, and acceptance. Regarding smell, participants evaluated the following attributes from 0 (no odor) to 5 (intense odor): black currant, carrot, citrus, flowery, fruit tea, fruity, honey, red currant, rose hip, sour, strawberry, sweetish, and berries of the forest. The taste also was rated from 0 to 5 for the attributes astringent, black currant, bitter, carrot, citrus, flowery, fruit tea, fruity, honey, red currant, rose hip, sour, sweet, and berries of the forest. Furthermore, each participant was asked about acceptance from 0 (I would not buy it) to 10 (very tasty, I would buy it).

### Gas chromatographic analysis

Flavor compounds were analyzed by gas chromatography–mass spectrometry–olfactometry (GC–MS–O) performed on an Agilent 7890B GC (Agilent Technologies, Santa Clara, USA) in combination with an Agilent 5977B MSD and an ODP 3 (GERSTEL GmbH & Co. KG, Mülheim a.d. Ruhr, Germany). It was equipped with a TDU 2, and a CIS 4 (all GERSTEL GmbH & Co. KG). A VF-WAXms and a DB-5 ms column were used (both 30 m · 250 µm · 0.25 µm; Agilent Technologies). 2 mL of samples were extracted at 24 °C for 1 h and 150 rpm with a Twister (PDMS, 10 mm, 0.5 mm film thickness, GERSTEL GmbH & Co. KG). Desorption was performed at 40 °C (0.5 min)/120 °C min^−1^/250 °C (10 °C) in the TDU and cryogenic focusing in CIS at − 70 °C (0.5 min)/12 °C s^−1^/250 °C (5 min). A constant flow of carrier gas (1.56 mL min^−1^, helium 5.0; Nipon Gasses GmbH, Hürth Germany) was equally split between MSD and ODP. The GC oven was heated to 40 °C (3 min)/5 °C min^−1^/240 °C (12 min). The MSD was operated in scan mode (source 230 °C, 70 eV, *m*/*z* 33–300).

GC-O analyses were performed by three participants (3 females, 24–28 years, non-smokers). Compounds were identified by comparison of retention indices (*RI*) according to van den Dool and Kraatz, recorded mass spectra, perceived odors, and in comparison to literature data (van den Dool and Kratz 1963). Fragments used for relative quantification are listed in Table S1 (Supporting information).

### Quantification of odor active compounds

GC analyses and SBSE extractions were performed as described above, but the GC was not equipped with an ODP, a TDU (GERSTEL GmbH & Co. KG) was used and the column flow was 1.2 mL min^−1^. Standard addition was performed with three steps by adding 0.5 mL standard solutions or 0.5 mL pure water to 2 mL of the beverage in 20 mL headspace vials (Table S2, Supporting information). 3,4-Dimethylbenzaldehyde, *trans*-nerolidol, phenylacetic acid, and terpinene-4-ol were quantified with splitless injection, and the other compounds with a split ratio of 50. Peak areas were determined using the fragments listed in Table S1 (Supporting Information) and plotted on the y-axis against the added mass. The intersection of the regression curve (*x*) was determined and the concentration (*c*) was determined with the used volume of the beverage (*V*) (Formula 1). The odor activity value (*OAV*) was determined by dividing the concentration (*c*) by odor thresholds (*OT*) (Formula 2) (Grosch 2001).

1 $$c=-x \cdot {V}^{-1}$$

2 $$OAV=c \cdot {OT}^{-1}$$

### Statistics

Samples were analyzed in random order. Averages, standard deviations, confidence intervals, and regression curves were determined with Excel. Sensory tests were performed by trained panelists and the statistical analysis of acceptance, odor and taste impressions were done with Excel using two-sample t-test for independent samples assuming different variances with *α* = 0.05 (Supporting Information, Table S3).

## Results and discussion

### Medium optimization

The pomace had a strong scent reminiscent of black currant and colored the medium intensely red. During autoclaving, odor and taste changed and the color of the medium turned towards brown (Figure S1). Firstly, the characteristic black currant-like odor disappeared and the scent was reminiscent of berries, fruit tea, and carrot juice.

The fermentation of the pomace-sugar-water suspension by *W. cocos* resulted in the formation of intense fruity and honey-like flavors. For medium optimization, the pomace content of 60 g L^−1^ was sequentially increased to 120 g L^−1^. A more intense taste and a denser mouth feeling, combined with a less watery impression resulted from higher pomace contents (Table S4, Supporting Information). Fermentations with 100 g L^−1^ or more showed a highly scented flowery-like odor, which was undesirable for a fruity beverage. The addition of sucrose prior to fermentation was essential for the aroma production. Reduced sugar content or the addition of sucrose after fermentation were both evaluated as less pleasant and reminiscent of fruit tea (Table S5, Supporting Information). Besides sucrose, the suitability of fructose and glucose was evaluated, and the odor profile was rated similarly but as less intense. The sour taste was weaker, and the beverage was overall rated as less tasty (Table S5, Supporting Information). Therefore, 80 g L^−1^ pomace and 58 g L^−1^ sucrose were chosen as the most suitable substrate for fermentation.

### pH and fruit acid formation during fermentation

An increased sour taste was observed during fermentation. The starting pH value of the cultures was 4.4 ± 0.2 after autoclaving which slowly decreased during the first hours of fermentation. The concentrations of citric acid changed barely during fermentation; 1000 ± 50 mg L^−1^ were detected in the non-fermented medium, and 1050 ± 10 mg L^−1^ in the fermented product after four days. The concentrations of oxalic acid increased strongly during fermentation from 8 ± 1 mg L^−l^ before fermentation to 1,990 ± 630 mg L^−1^ after four days. Oxalic acid is the simplest dicarboxylic acid and occurs in several plants like spinach and rhubarb, animals, and bacteria (Noonan and Savage 1999). It has a pleasant, characteristic, and astringent taste (Noonan and Savage 1999). Cultures with a pH value of 3.5 revealed a pleasantly sour taste. The pH value was thus used as an indicator for the time of harvesting. All determined oxalic acid concentrations for fermented beverages with pH values of ≥ 3.5 were below 300 mg L^−1^_._

### Upscaling and pilot plant processing

To test whether the produced beverage is suitable for a large-scale production, the fermentation was scaled up in shaking flasks. All cultures showed the characteristic odor and taste observed in the small-scale fermentations. The time of harvesting as defined by reaching a pH value of 3.5 ± 0.1 led to highly comparable impressions and proved the robustness of the pH value as an indicator for the termination of the fermentation process. Depending on the respective culture volume, the final pH value of 3.5 was reached after different fermentation periods. In 0.2 L in 0.5 L EK, a pH value of 3.5 was reached after 2.5 days, in 0.8 L in 2 L EK after 3 days, and in 2 L in 5 L EK after 4.5 days. Furthermore, the fermentation was performed in a fermenter with a volume of 4 L, in which a pH of 3.5 was reached after 4 days (Supporting Information, Figure S2). The flavor profile and the detected aroma compounds were highly similar to those observed in the smaller volumes (Supporting Information, Figure S3). This indicated that the fermentation process could be easily adapted to a bigger scale.

For pilot scale processing, 15 L of the fermented drink was produced using 5 L EF with 2 L culture volume. The oxalic acid content of the non-processed drink directly after fermentation was 232 ± 3 mg L^−1^. During the filtration, the color changed to a lighter, intense red color. The beverage retained the pleasant sour–sweet character and 935 ± 20 mg L^−1^ of citric acid and 192 ± 2 mg L^−1^ oxalic acid were determined. The unique sour taste of citric acid and the sour and astringent taste of oxalic acid matched the impression of the beverage very well. Especially oxalic acid played an important role for the fruity and fresh taste of the fermented product.

### Sensory analysis of the beverages

**Fig. 2:**
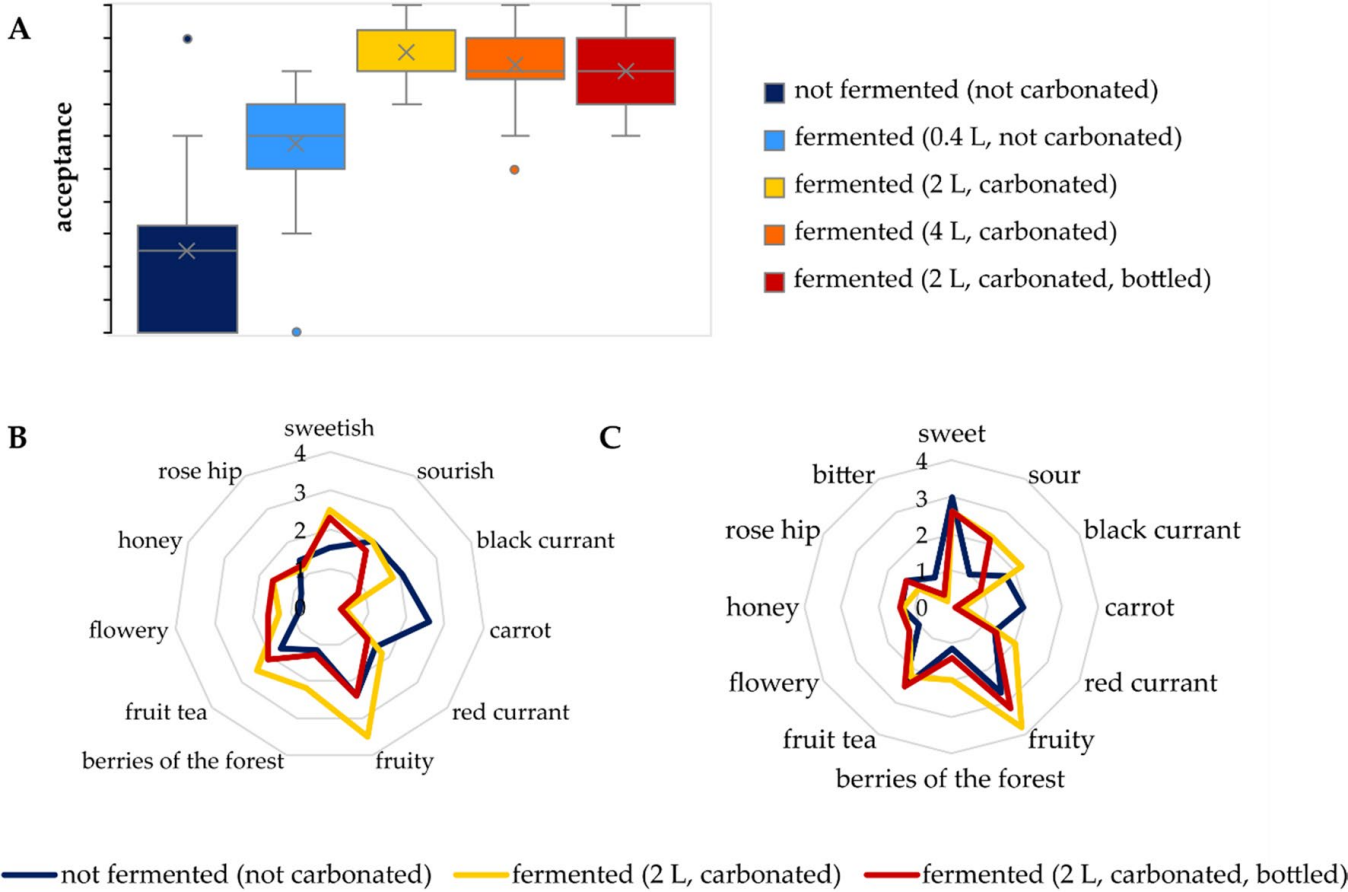
**A** Overall acceptance from 0 (“I would not buy it”) to 10 (“very tasty, I would buy it”) of the medium (dark blue), the fermented beverage with increasing culture volume in shaking flasks (light blue, yellow), produced in the fermenter (orange) and the bottled beverage (red); description of the odor **B** and the taste **C,** comparing the non-fermented medium (dark blue) with the freshly fermented product (yellow) and the bottled beverage (red) with 0 (no perceived odor/taste) and 5 (very intensive odor/taste), *n* = 20 (Color figure online)

Prior to the fermentation process, the participants of the panel rated the medium as not tasty enough to buy it and evaluated it with 2.5 ± 2.4 of 10 (Fig. 2A). After fermentation and carbonization, the acceptance increased significantly. The bottled drink, which was additionally filtered and pasteurized, was rated similarly high (8.0 ± 1.4 of 10).

The non-fermented substrate had a strong impression reminiscent of carrots, which was evaluated as unpleasant (Fig. 2B). The fermentation with *W. cocos* changed the odor profile and removed this unpleasant note. Furthermore, the perception of the impressions fruity, sweetish, berries of the forest, and honey increased. After processing, the profile remained similar, but the odor impression intensities slightly decreased for the attributes fruit tea, fruity, berries of the forest, and black currant. The taste profile also changed during fermentation (Fig. 2C). Similar to the odor, the carrot-like impression was perceived before fermentation and disappeared due to bioconversion. Regarding the taste, the attributes sour, black currant, fruity, and berries of the forest increased. Between the bottled and the non-processed sample, the differences in taste were weaker in comparison to the odors. The sour taste induced by oxalic acid correlated well with the concentrations of the fruit acids (Table S6, Supporting information). The sweet taste was comparable in all products. As indicated in Table S7 (Supporting Information), the sugar content did not change strongly during the processing steps. The bottled beverage fermented with *W. cocos* had a sugar content of 49 ± 1 g L^−1^. In particular, 2.7 ± 0.9 g L^−1^ glucose, 4.8 ± 0.8 g L^−1^ fructose, and 42 ± 0.22 g L^−1^ sucrose were detected. Several beverages with a reduced sugar content have been launched in the past. The following sugar contents were found by comparing 183 beverages on the German market: 51 ± 9 g L^−1^ in sugar-reduced lemonades, 62 ± 14 g L^−1^ in *Fassbrause*-varieties (a traditional German fermented drink), 86 ± 17 g L^−1^ in classical lemonades, and 92 ± 9 g L^−1^ in soft drinks. The sugar amount of the fermented pomace beverage is comparable to those of sugar-reduced lemonades and *Fassbrause*, which both have high consumer acceptance.

### Aroma compounds

**Fig. 3:**
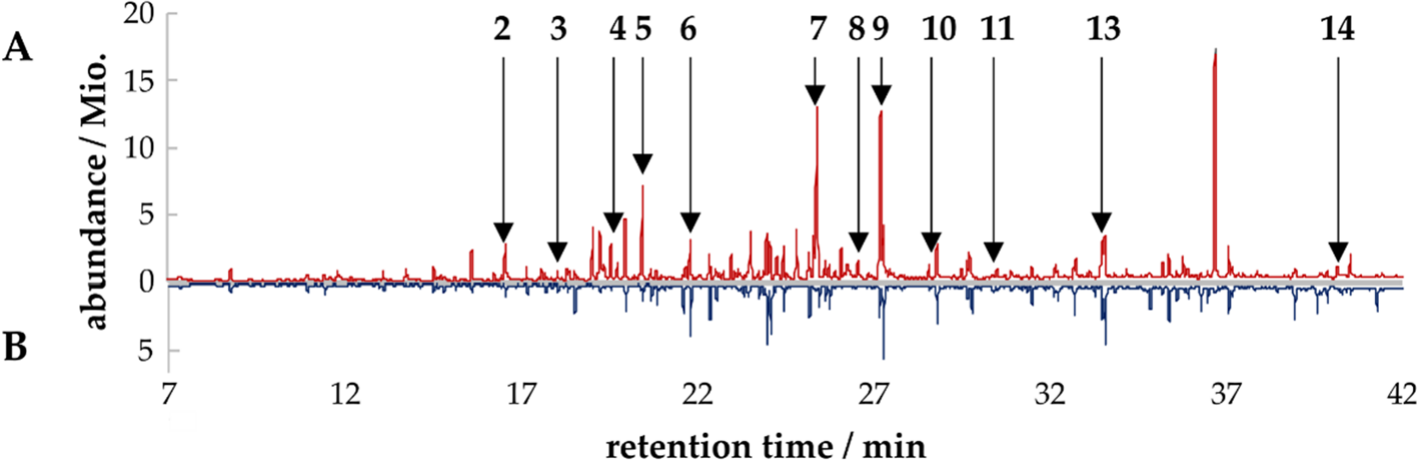
SBSE-GC–MS-chromatogram (scan, *m*/*z* 33–300) of the **A** bottled beverage and the **B** non-fermented substrate with identified odor active compounds of the bottled beverage 2-nonanone **2**, 1-octen-3-ol **3**, 2-nonanol **4**, linalool **5**, terpinene-4-ol **6**, methyl phenylacetate **7**, 3,4-dimethylbenzaldehyde **8**, geraniol **9**, 2-phenylethanol **10**, *trans*-nerolidol **11**, eugenol **13** and phenylacetic acid **14**

**Fig. 4:**
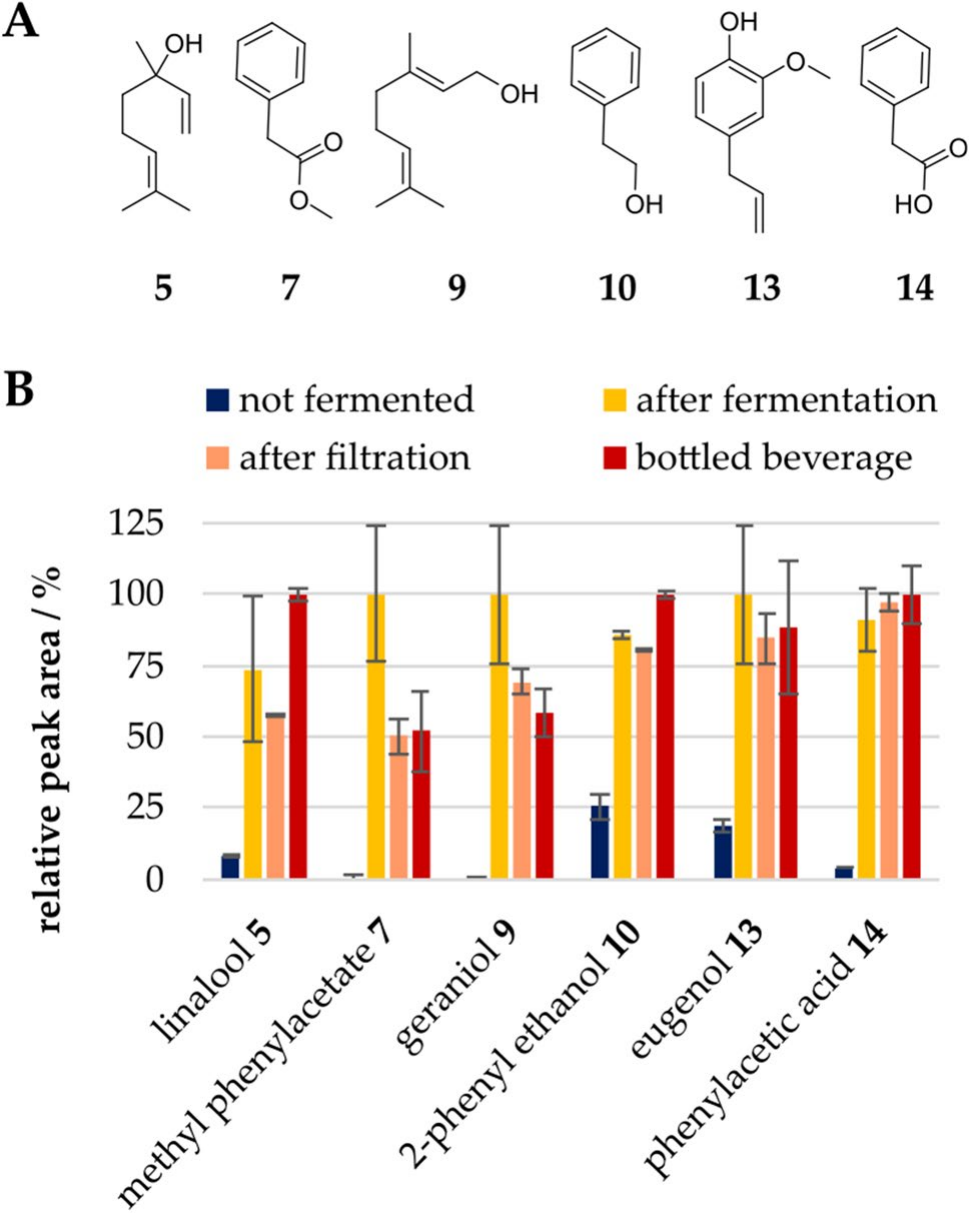
**A** Structural formula of linalool **5**, methyl phenylacetate **7**, geraniol **9**, 2-phenylethanol **10**, eugenol **13**, phenyl acetic acid **14**, **B** relative SBSE-GC–MS peak areas of odor-active compounds (OAV > 1) before the fermentation (blue), after the fermentation (yellow), after filtration (orange), and in the bottled beverage (red) (Color figure online)

GC–MS–O analyses of the non-fermented and the fermented beverage were performed to investigate the origin of the honey-like, flowery, and fruity odor. The chromatogram of the fermented beverage included several compounds, which were not detected in the non-fermented medium (Fig. 3A, B). To detect aroma compounds, GC-O measurements were performed with three participants, and 14 odor impressions were detected by at least two participants (Table 1). The identified compounds were quantified by means of standard addition. All regression curves had a coefficient of determination > 0.98. The concentrations of linalool **5**, geraniol **7**, phenylacetic acid **14**, methyl phenylacetate **7**, eugenol, and 2-phenylethanol **10** exceeded the odor thresholds (Table 2, Fig. 4A). Therefore, these compounds do likely influence the overall flavor impression. 1-Octen-3-ol **3**, 2-nonanol **4**, terpinene-4-ol **6**, 2-nonanone **2**, and *trans*-nerolidol 11 showed odor activity values below 0.1, and therefore most likely did not contribute to the overall aroma. Methyl phenylacetate **7** and phenylacetic acid **14** are known for their intense honey-like scent, and geraniol **9**, 2-phenylethanol **10**, and linalool **5** for their flowery and citrus-like notes. Eugenol **13** has a sweetish odor and is characteristic of clove. These compounds were absent in the non-fermented beverage or only detected in minor amounts (Fig. 4B). During the processing of the fermented beverage, the intensities of methyl phenylacetate **7** and geraniol **9** decreased, whereas the areas of the other compounds stayed constant. The compounds which define the pleasant aroma of the beverage are not typical for black currants. 4-Methoxy-2-methyl-2-butanthiol, (3*Z*)-hexenal, 1-octen-3-one, 1,8-cineol, and ethyl butanoate typically characterize the odor of black currants due to their high odor activity values. (Jung et al. 2017). 4-Methoxy-2-methyl-2-butanthiol has a characteristic black currant like odor but is also ascribed as a catty urine smell in higher concentrations. It was neither detected by sensory evaluation nor by GC–MS measurements in the medium or the fermented cultures. This characteristic flavor compound of black currants is probably lost during autoclaving. (3*Z*)-Hexenal is known to be removed by freezing and was also not detected (Jung et al. 2017). Odor impression 1 “mushroom” (Table 1) has the same odor and retention index as 1-octen-3-one. Because of its very low odor threshold (0.03 -1.12 ng L^−1^ air), the nose could be more sensitive than the MS detector (Guth and Grosch 1990). Therefore, the odor impression could be explained by small amounts of 1-octen-3-one. 1,8-Cineol with its terpenic odor and ethyl butanoate with a pineapple-like odor were detected by GC–MS in the medium and the fermented beverage. During GC-O measurements, they were perceived less intensely than the compounds listed in Table 1. Overall, the beverage is characterized by a unique flavor composition, which can hardly be compared to the odor profile of fruit juices or other known beverages. The comparison with fermentations without pomace showed that the relative peak areas of the odor-active compounds differed from those of cultures without pomace (Figure S4, Supporting Information). Although the overall flavor impression does not resemble that of black currant, the pomace represents a rich source of nutrients to enable the formation of the flavor compounds by *W. cocos*.

**Table 1:** identified compounds with retention indices (*RI*), odor impressions perceived by GC-O, and parameters of identification: concordance of MS with NIST database (MS), *RI* and odor description (O) with published data, with authentic standard (STD), and matching odor impression, with n.i. = compound not identified, * = calculated with the retention time of the odor impression

	Substance	*RI*_VF-WAXms_	*RI*_DB-5 ms_	Identification	Odor impression
1	n.i.	1305*	–	–	Mushroom
2	2-Nonanone	1392	1093	*RI*, MS, STD, O	Flowery, burnt
3	1-Octen-3-ol	1454	984	*RI*, MS, STD, O	Earthy, fruity
4	2-Nonanol	1522	1103	*RI*, MS, STD O	Flowery, earthy, mushroom
5	Linalool	1551	1102	*RI*, MS, STD, O	Flowery, citrus, blueberry
6	Terpinen-4-ol	1607	1185	*RI*, MS, STD, O	Woody, fatty, flowery
7	Methyl phenylacetate	1767	1178	*RI*, MS, STD, O	Beeswax, honey
8	3,4-Dimethyl benzaldehyde	1820	1218	*RI*, MS, STD, O	Fruity, sweetish, peach
9	Geraniol	1851	1253	*RI*, MS, STD, O	Flowery, earthy
10	2-Phenylethanol	1917	1115	*RI*, MS, STD, O	Flowery, rose, sweetish
11	*Trans*-nerolidol	2037	1562	*RI*, MS, STD, O	Sweetish, cocos, woodruff
12	n.i.	2132*	–	–	Woody, green, sourish
13	Eugenol	2173	1354	*RI*, MS, STD, O	Clove, sweetish
14	phenylacetic acid	2565	1249	*RI*, MS, STD, O	Beeswax, honey

**Table 2:** concentrations, odor thresholds, and odor activity values of the identified flavor compounds with (a) (Schuh and Schieberle 2006), (b) (Ruisinger and Schieberle 2012), (c) (Bonvehí 2005) (d) (Pino and Mesa 2006), (e) (Schmidberger and Schieberle 2020), (f) (Moio et al. 2000), and n.a. = not available

	Concentration/µg L^−1^	Odor threshold/µg L^−1^	OAV
Linalool	22.7 ± 2.9	0.6 (a)	37.8 ± 4.8
Geraniol	42.0 ± 1.1	3.2 (a)	13.1 ± 0.4
Phenylacetic acid	948 ± 98	135 (b)	7.0 ± 0.7
Methyl phenylacetate	94.1 ± 7.1	60 (c)	1.6 ± 0.1
Eugenol	5.86 ± 0.21	6 (d)	1.0 ± 0.1
2-Phenylethanol	73.8 ± 14.9	89 (b)	0.8 ± 0.2
1-Octen-3-ol	2.04 ± 1.63	45 (e)	0.05 ± 0.04
2-Nonanol	1.65 ± 0.17	20 (f)	0.08 ± 0.01
Terpinene-4-ol	6.17 ± 0.25	130 (d)	0.02 ± 0.01
2-Nonanone	0.80 ± 0.08	75 (f)	0.01 ± 0.01
*Trans*-nerolidol	0.10 ± 0.07	250 (d)	< 0.01
3,4-Dimethyl benzaldehyde	1.77 ± 0.25	n.a.	n.a.

*W. cocos* has been used to ferment several substrates in the past, and some of the odor-active compounds detected in this study have also been reported previously. Sommer et al. (2021) fermented black currant pomace with *W. cocos* in solid-state fermentation. Supplementation of the solid-state cultures with sodium aspartate led to a wild-strawberry like odor. Linalool, geraniol, methyl anthranilate, and 2-aminobenzaldehyde were revealed to be key aroma compounds in recombination experiments. Rigling et al. (2021a) fermented brewed green tea with *W. cocos*, and the odor of the beverage was described as jasmine-like. Besides methyl anthranilate, linalool, geraniol, 2-phenylethanol and methyl phenylacetate were detected as aroma-relevant. Wang et al. (2022) fermented okara with *W. cocos*, and the fungus successfully reduced the beany flavor and produced aroma compounds like 1-octen-3-ol, benzaldehyde, 2-undecanone, geraniol, linalool, and methyl phenylacetate (Wang et al. 2022). Overall, the biotransformation properties of *W. cocos* revealed an enormous potential for the production of pleasant aromas. Various side streams of the juice industry may thus be upcycled to a wide range of fermented beverages.

### Toxicological and commercial aspects

The sclerotium of *W. cocos* is a traditional tea ingredient in Chinese medicine. No acute or chronic toxicity was described for a dosage of 18 g sclerotium d^−1^ (Wang et al. 2013). From the beverage developed in this study, the fungal mycelium is removed by filtration, and the beverage is pasteurized prior to consumption. In accordance with Regulation (EU) 2015/2283, the produced beverage will probably be classified as a novel food, and is therefore subject to an approval procedure in the European Union. However, as no harmful effects have been described for *W. cocos*, the fungal fermentation is highly promising (Wang et al. 2013). A potentially toxicologically relevant compound in the beverage is oxalic acid, which is suspected to cause kidney stones consisting of calcium oxalate (Noonan and Savage 1999). Cell culture studies showed a correlation of oxalic acid concentration and reactive oxygen species (Moryama et al. 2005). In a recent review, the interaction of calcium oxalate and renal epithelial cells in in vitro cell culture models has been discussed, and calcium oxalate has been reported to trigger epithelial-mesenchymal transition and carcinogenic features in renal cells (Petrovic et al. 2021; Peerapen et al. 2022). A typical daily intake of approximately 220 mg oxalate has been reported in the literature (Crivelli et al. 2020), however, the contribution of the oral intake of oxalic acid for the formation of kidney stones is not yet fully understood. The brown-rot fungus *W. cocos* is known to decrease the pH of the culture medium by producing oxalic acid (Espejo and Agosin 1991). A concentration of 192 ± 2 mg L^−1^ oxalic acid was detected in the processed beverage. Other foods contain much higher amounts of oxalic acid. For instance, spinach contains 3200–12,600 mg kg^−1^ and the oxalic acid concentrations of rhubarb range from 2750 to13,360 mg kg^−1^ (Noonan and Savage 1999). Rhubarb juice is also a popular drink, which contains approximately 3400 mg L^−1^ oxalic acid (Will and Dietrich 2013). The oxalic acid concentration in rhubarb juice exceeds the concentration of the pomace based fermented beverage by a factor of 18. Regarding the oxalic acid concentration, the developed beverage seems to be suitable for occasional human consumption.

The recycling of pomace for the production of a fermented beverage is highly sustainable and could reduce the need for classical spritzer and lemonades. As 29% of the berry mass remains as pomace, 3.6 L of the fermented beverage can be produced from the side streams of one kg berries. Therefore, resources may be saved as less cultivation area is needed, less product is burnt and a healthy and palatable diet is enabled (May and Guenther 2020; Struck et al. 2016).

## Conclusion

We have found a highly sustainable method to reuse black currant pomace for human nutrition by the use of the fungus *W. cocos*. The fermented beverage was described as highly tasty and has the potential to be easily scaled up. The substrate-fungus combination led to a pleasant, unique flavor profile. As the fungus is described as edible and known as a natural drug, it has a great potential not only as a biocatalyst for flavor and fruit acid formation, but also as a functional drink. Further research regarding the nutritional and potential health benefits could be highly interesting.

## Supplementary Information

Below is the link to the electronic supplementary material.

Supplementary file 1 (DOCX 822 kb)

## Data Availability

The datasets analysed during the current study are available from the corresponding author on reasonable request.

## Abbreviations

CIS: Cold injection system
EF: Erlenmeyer flask
GC–MS–O: Gas chromatography–mass spectrometry–olfactometry
MEA: Malt extract agar
*n*: Sample number
n.i.: Not identified
NIST: National institute for standards and technologies
ODP: Olfactory detection port
*RI*: Retention index
rpm: Rounds per minute
STD: Standard
TDU: Thermal desorption unit
TIC: Total ion count
*W. cocos*: *Wolfiporia cocos*
