# Supplementary material for: Upcycling of black currant pomace for the production of a fermented beverage with Wolfiporia cocos
Source: J Food Sci Technol. 2023 Feb 6;60(4):1313–22. doi: 10.1007/s13197-023-05677-4 (PMC10020415; doi:10.1007/s13197-023-05677-4)
Supplement: Supplementary file 1 — (DOCX 822 kb) [file 13197_2023_5677_MOESM1_ESM.docx]

# SUPPORTING INFORMATION

## Upcycling of black currant pomace for the production of a fermented beverage with *Wolfiporia cocos*


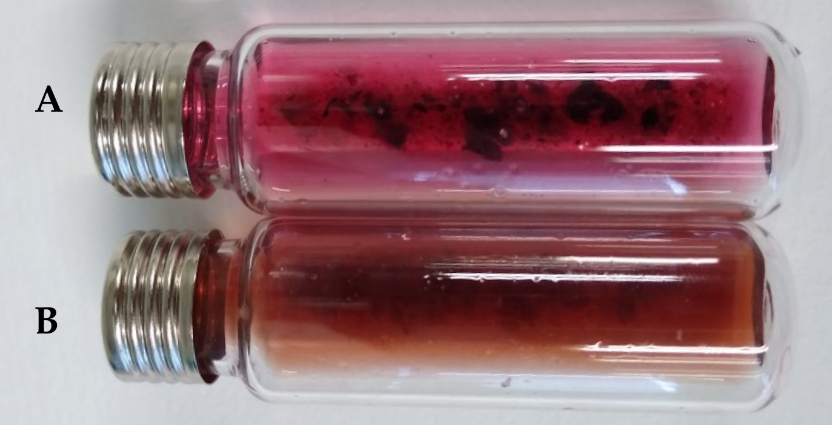


Figure S 1 20 mL headspace vial filled with 5 mL medium (80 g L^-1^ pomace, 58 gL^-1^ sucrose) **A** prior to autoclaving and **B** after autoclaving


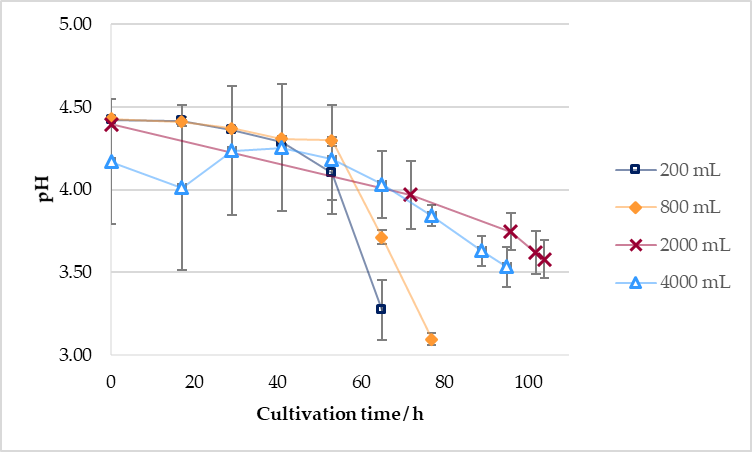


Figure S 2 pH development during fermentation of 80 g L^-1^ pomace and 60 g L^-1^ sucrose in shaking flasks with culture volumes of 200 mL, 800 mL, and 2000 mL and the fermenter 4000 mL (*n* = 3)


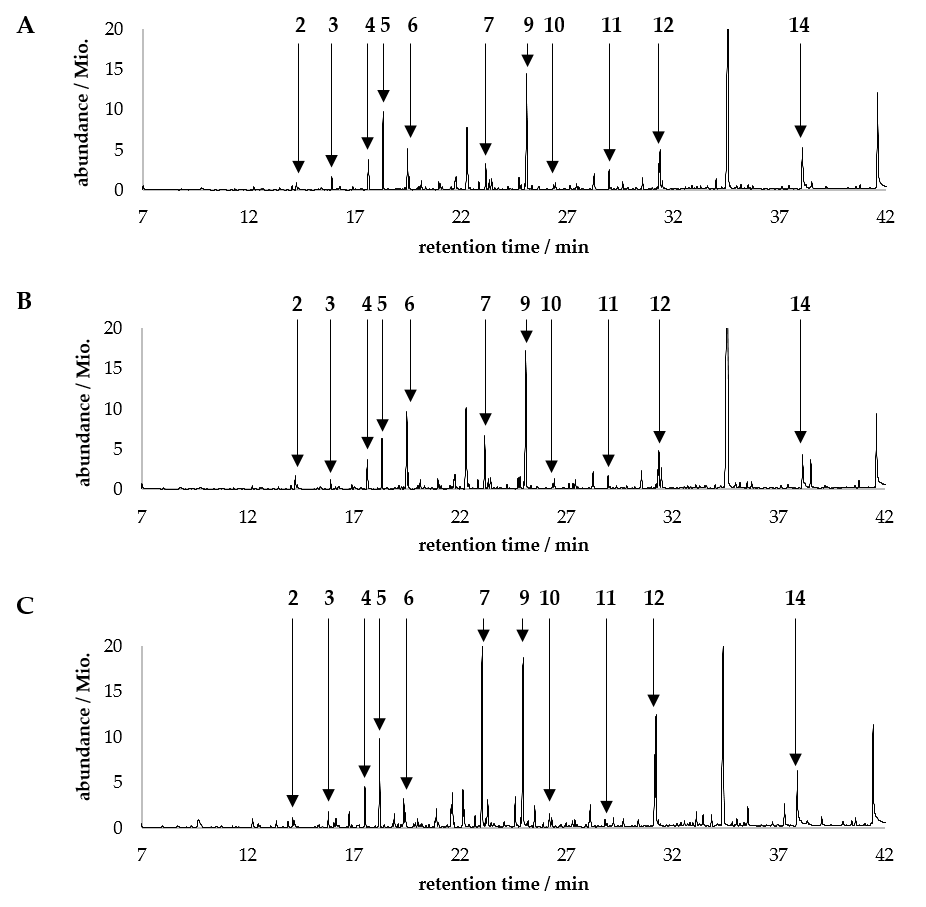


Figure S 3 SBSE-GC-MS chromatograms (scan, m/z 33-300) of the supernatant harvested at pH 3.5 ± 0.1 with labeled identified flavor compounds 2-nonanone **2**, 1-octen-3-ol **3**, 2-nonanol **4**, linalool **5**, terpinene-4-ol **6**, methyl phenylacetate **7**, geraniol **9**, 2-phenylethanol **10**, *trans*-nerolidol **11**, eugenol **13,** and phenylacetic acid **14** in up-scaled flask sizes **A** 200 mL in 500 mL Erlenmeyer flasks (EF), **B** 800 mL in 2 L EF, and **C** 4L in a 7.5 L fermenter


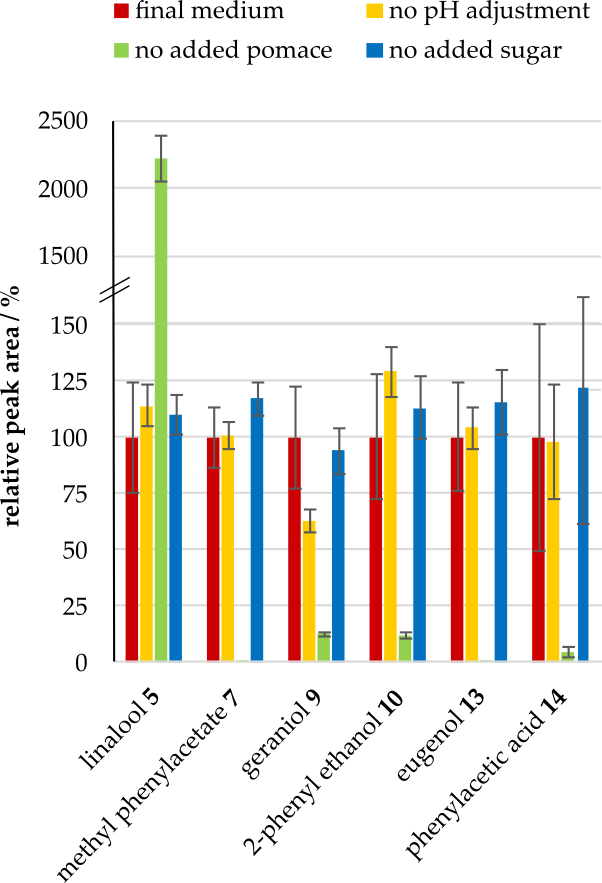


Figure S 4 relative peak areas of odor active compounds in relation to the beverage of the final medium after fermentation (red) for fermented medium without pH adjusting before fermentation (yellow), in fermented medium of pure sucrose without pomace, pH 4.4 (green), and in fermented medium of pomace, pH 4.4 without sugar (blue) of pure determined by SBSE-GC-MS.

Table S 1 *m*/*z* of the fragments used as quantifier and qualifier of odor-active compounds

|  | ***m*/*z* (quantifier)** | ***m*/*z* (qualifier 1)** | ***m*/*z* (qualifier 2)** |
| --- | --- | --- | --- |
| 2-nonanone | 58 | 43 | 71 |
| 1-octen-3-ol | 57 | 72 | 43 |
| 2-nonanol | 45 | 69 | 98 |
| linalool | 71 | 93 | 121 |
| terpinen-4-ol | 71 | 93 | 111 |
| methyl phenylacetate | 91 | 92 | 122 |
| 3,4-dimethyl benzaldehyde | 133 | 134 | 105 |
| geraniol | 69 | 41 | 123 |
| 2-phenylethanol | 91 | 92 | 122 |
| *trans*-nerolidol | 69 | 93 | 107 |
| eugenol | 164 | 149 | 131 |
| phenylacetic acid | 91 | 136 | 65 |

Table S 2 concentrations (*c*) used for standard addition of the flavor compounds.

|  | **c_1_ / µg L^-1^** | **c_2_ / µg L^-1^** | **c_3_ / µg L^-1^** |
| --- | --- | --- | --- |
| 2-nonanone | 4.0 | 12.0 | 20.0 |
| 1-octen-3-ol | 13.5 | 40.5 | 67.5 |
| 2-nonanol | 4.0 | 12.0 | 20.0 |
| Linalool | 100 | 300 | 500 |
| terpinen-4-ol | 10 | 30 | 50 |
| methyl phenylacetate | 275 | 826 | 1377 |
| 3,4-dimethyl benzaldehyde | 2.0 | 6.0 | 10.1 |
| geraniol | 25 | 75 | 125 |
| 2-phenylethanol | 60 | 180 | 300 |
| *trans*-nerolidol | 2.0 | 6.0 | 10.0 |
| eugenol | 10 | 30 | 50 |
| phenylacetic acid | 1000 | 3000 | 5000 |

Table S 3 parameters of the statistical analysis of two-sample t-test for independent samples assuming different variances with *α* = 0.05 comparing the non-fermented medium with the bottled beverage with the averages, standard deviations (σ), t-statistics values, and P(T **≤** t) both sides

|  | **non-fermented beverage** | | **bottled beverage** | | **statistical parameters** | |
| --- | --- | --- | --- | --- | --- | --- |
|  | **average** | **σ** | **average** | **σ** | **t-statistic** | **P(T ≤ t)** |
| overall acceptance | 2.5 | 2.5 | 8.0 | 1.4 | -8.78 | 8.6 10^-10^ |
| **ODOR** | | | | | | |
| sweetish | 1.6 | 1.2 | 2.3 | 1.3 | -1.78 | 0.082 |
| sourly | 2.0 | 1.5 | 1.7 | 1.1 | 0.84 | 0.408 |
| black currant | 2.1 | 1.7 | 0.8 | 0.9 | 3.01 | 0.004 |
| carrot | 2.6 | 1.9 | 0.3 | 0.6 | 5.23 | 3.0 10^-5^ |
| red currant | 1.6 | 1.6 | 1.3 | 1.2 | 0.57 | 0.572 |
| fruity | 2.4 | 1.8 | 2.4 | 1.3 | 0.00 | 1.000 |
| berries of the forest | 1.2 | 1.3 | 1.3 | 1.1 | -0,26 | 0.792 |
| fruit tea | 1.7 | 1.4 | 2.1 | 1.3 | -1.03 | 0.308 |
| flowery | 0.8 | 1.1 | 1.6 | 1.2 | -2.06 | 0.046 |
| honey | 0.8 | 1.1 | 1.6 | 1.4 | -2.01 | 0.050 |
| rose hip | 1.5 | 1.4 | 1.3 | 1.3 | 0.35 | 0.728 |
| **TASTE** | | | | | | |
| sweet | 3.0 | 1.3 | 2.6 | 0.8 | 1.19 | 0.242 |
| sour | 1.0 | 1.2 | 2.1 | 0.9 | -3.24 | 0.002 |
| black currant | 1,7 | 1.5 | 0.9 | 0.9 | 2.12 | 0.041 |
| carrot | 2.0 | 1.6 | 0.1 | 0.2 | 5.14 | 4.9 10^-5^ |
| red currant | 1.4 | 1.4 | 1.4 | 1.4 | -0.11 | 0.910 |
| fruity | 2.7 | 1.6 | 3.2 | 1.0 | -1.09 | 2.037 |
| berries of the forest | 1.2 | 1.2 | 1.4 | 1.3 | -0.63 | 0.531 |
| fruit tea | 2.5 | 1.4 | 2.5 | 1.4 | 0.00 | 1.000 |
| flowery | 1.0 | 1.3 | 1.3 | 1.1 | -0.79 | 0.432 |
| honey | 1,4 | 1.2 | 1.4 | 1.2 | 0.00 | 1.000 |
| rose hip | 1.4 | 1.4 | 1.4 | 1.2 | 0.12 | 0.904 |
| bitter | 0.9 | 1.2 | 0.4 | 0.6 | 1.65 | 2.048 |

Table S 4 odor and taste description, pH, and oxalic acid concentrations of non-fermented (d0), and fermented beverages for three days (d3) and four days (d4), (*n* =2)

|  | **Odor** | **taste** | **pH** | **oxalic acid / mg L^-1^** |
| --- | --- | --- | --- | --- |
| **60 g L^-1^ pomace, 58 g L^-1^ sucrose** | | | | |
| d0 | carrot juice, slightly berry-like | very sweet, neutral | - | - |
| d3 | flowery, fruity, slightly carrot, sweetish | sweet, sour, honey, fruity | 2.21 ± 0.02 | 1725 ± 103 |
| d4 | intensively flowery, less fruity, more sweetish | very sour, honey, fruity | 2.10 ± 0.05 | 2062 ± 153 |
| **80 g L^-1^ pomace, 58 g L^-1^ sucrose** | | | | |
| d0 | carrot juice, fruit tea | fruit tea, carrot | - | - |
| d3 | fruity, sweetish, cherry | fruity, sweet, sour, berries of the forest | 2.60 ± 0.34 | 1472 ± 398 |
| d4 | fruity, sweetish, red currant | fruity, very sour, red currant, berries of the forest | 2.36 ± 0.30 | 1974 ± 580 |
| **100 g L^-1^ pomace, 58 g L^-1^ sucrose** | | | | |
| d0 | intensively carrot, fruit tea | intense fruit tea, carrot | - | - |
| d3 | less fruity, intensively honey, flowery | fruit tea, sweet, sour, honey | 2.75 ± 0.17 | 1705 ± 106 |
| d4 | fruit tea, honey, flowery | sour, astringent, sweet, bitter | 2.50 ± 0.15 | 2066 ± 259 |
| **120 g L^-1^ pomace, 58 g L^-1^ sucrose** | | | | |
| d0 | intensively carrot, fruit tea | intense fruit tea, carrot | - | - |
| d3 | intensively flowery, fruity, berries | very sweet, flowery, astringent | 3.24 ± 0.23 | 1051 ± 377 |
| d4 | intensively flowery, honey, fruity, berries | sweet, sour, flowery | 2.82 ± 0.10 | 1738 ± 225 |

Table S 5 odor description and taste description of non-fermented-medium and fermented beverages with a reduced sucrose content, with addition of sucrose after fermentation and with glucose or fructose as sugar source (*n* = 5)

| **Odor** | **taste** | **pH** | **time / h** |
| --- | --- | --- | --- |
| **80 g L^-1^ pomace, 58 g L^-1^ sucrose - not fermented** | | | |
| carrot, hay, sweetish, sourly | sweet, carrot, fruit tea, tart, honey | 4.18 ± 0.20 | 77 |
| **80 g L^-1^ pomace, 58 g L^-1^ sucrose added after fermentation** | | | |
| tart, fruit tea, carrot, rose hip, sweetish | sweet, astringent, sour, fruit tea, berries of the forest, carrot | 3.21 ± 0.10 | 65 |
| **80 g L^-1^ pomace, 44 g L^-1^ sucrose – fermented** | | | |
| fruit tea, neutral, Earl Grey, fruity, sweetish | sour, sweet, neutral, fruit tea, Earl Grey, bitter, astringent | 3.30 ± 0.06 | 65 |
| **80 g L^-1^ pomace, 84 g L^-1^ glucose – fermented** | | | |
| tart, bland, tea, strawberry, sweetish, rose hip | rose hip, fruit tea, metallic, astringent, sour bitter, sweet | 3.51 ± 0.15 | 77 |
| **80 g L^-1^ pomace, 51 g L^-1^ fructose – fermented** | | | |
| bland, hey, tea, rose hip, sourly | sweet, astringent, metallic, fruit tea, strawberry, citrus | 3.44 ± 0.06 | 65 |

Table S 6 concentrations of fruit acids during fermentation and processing of the beverage

|  | **citric acid / mg L^-1^** | **oxalic acid / mg L^-1^** |
| --- | --- | --- |
| before fermentation | 986 ± 25 | 8 ± 1 |
| after fermentation | 1054 ± 13 | 233 ± 3 |
| after enzymatic treatment | 980 ± 1 | 229 ± 2 |
| after filtration | 914 ± 1 | 189 ± 1 |
| bottled beverage | 935 ± 20 | 192 ± 2 |

Table S 7 sugar content during fermentation and processing of the beverage

|  | **glucose / g L^-1^** | **fructose / g L^-1^** | **sucrose / g L^-1^** | **sugar / g L^-1^** |
| --- | --- | --- | --- | --- |
| before fermentation | 1.8 ± 1.0 | 3.0 ± 0.3 | 48.4 ± 3.0 | 53.2 ± 2.4 |
| after fermentation | 1.7 ± 2.1 | 3.1 ± 1.8 | 47.3 ± 3.8 | 52.1 ± 7.6 |
| after enzymatic treatment | 5.6 ± 0.5 | 7.0 ± 0.3 | 36.4 ± 1.5 | 49.0 ± 2.3 |
| after filtration | 1.1 ± 0.4 | 3.1 ± 0.4 | 44.7 ± 2.3 | 48.9 ± 3.0 |
| bottled beverage | 2.7 ± 0.9 | 4.8 ± 0.8 | 42. 0 ± 2.7 | 49.4 ± 4.5 |
